# A universal harm-minimisation approach to preventing psychostimulant and cannabis use in adolescents: a cluster randomised controlled trial

**DOI:** 10.1186/1747-597X-9-24

**Published:** 2014-06-18

**Authors:** Laura Elise Vogl, Nicola Clare Newton, Katrina Elizabeth Champion, Maree Teesson

**Affiliations:** 1 National Drug and Alcohol Research Centre, University of New South Wales, 22-32 King Street, Randwick 2031, New South Wales, Australia; 2 National Health and Medical Research Council Centre for Research Excellence in Mental Health and Substance Use, National Drug and Alcohol Research Centre, University of New South Wales, 22-32 King Street, Randwick 2031, New South Wales, Australia

**Keywords:** School, Harm-minimisation, Computer-based, Universal, Psychostimulant, Cannabis, Prevention

## Abstract

**Background:**

Psychostimulants and cannabis are two of the three most commonly used illicit drugs by young Australians. As such, it is important to deliver prevention for these substances to prevent their misuse and to reduce associated harms. The present study aims to evaluate the feasibility and effectiveness of the universal computer-based Climate Schools: Psychostimulant and Cannabis Module.

**Methods:**

A cluster randomised controlled trial was conducted with 1734 Year 10 students (mean age = 15.44 years; SD = 0.41) from 21 secondary schools in Australia. Schools were randomised to receive either the six lesson computer-based Climate Schools program or their usual health classes, including drug education, over the year.

**Results:**

The Climate Schools program was shown to increase knowledge of cannabis and psychostimulants and decrease pro-drug attitudes. In the short-term the program was effective in subduing the uptake and plateauing the frequency of ecstasy use, however there were no changes in meth/amphetamine use. In addition, females who received the program used cannabis significantly less frequently than students who received drug education as usual. Finally, the Climate Schools program was related to decreasing students’ intentions to use meth/amphetamine and ecstasy in the future, however these effects did not last over time.

**Conclusions:**

These findings provide support for the use of a harm-minimisation approach and computer technology as an innovative platform for the delivery of prevention education for illicit drugs in schools. The current study indicated that teachers and students enjoyed the program and that it is feasible to extend the successful Climate Schools model to the prevention of other drugs, namely cannabis and psychostimulants.

**Trial registration:**

Australian and New Zealand Clinical Trials Registry ACTRN12613000492752.

## Background

Psychostimulants and cannabis have emerged as the most commonly used illicit drugs in Australia [1] and are two of the three most commonly used illicit drugs by young Australians (the other being inhalants) [2]. Early initiation to drug use has been associated with a range of negative consequences including substance use disorders, co-morbid mental health problems, juvenile offending, impaired educational performance and early school drop-out, resulting in negative impacts on both current functioning and future life options [3-5]. As such, the need for effective prevention programs is clear and schools offer the ideal location to deliver them. School-based prevention programs are ideally placed to access a vulnerable population of young people before significant drug use problems develop [6-10]. A number of school-based drug prevention programs do exist, but the outcomes of these programs are often compromised by implementation failure [11-14].

The current study draws on previous research on universal school-based prevention programs known as Climate Schools. The Climate Schools programs are underpinned by a harm-minimisation framework, use cartoon storylines to engage and maintain student interest, and are designed to overcome problems with implementation through the use of computer technology. Computer-based programs can be implemented with a higher degree of fidelity as they consist of pre-programmed content, meaning that the core components of the program are maintained [15], as teachers are unable to make deletions or additions [16]. To date, two Climate Schools programs have been developed and evaluated. The six lesson Climate Schools: Alcohol Module aims to address alcohol misuse and related harms in Year 8 students (13–14 year olds) [17] and has been evaluated through two randomised controlled trials (RCTs) [18,19]. The module was found to significantly increase alcohol-related knowledge, decrease positive expectancies about alcohol and reduce average alcohol consumption, alcohol-related harms and the frequency of drinking to excess among Australian adolescents [19]. The Climate Schools: Alcohol and Cannabis Module, which consists of a further six lessons, was designed to act as a booster to the Alcohol Module whilst also delivering new information on cannabis. Together the modules make up the 12-lesson Climate Schools: Alcohol and Cannabis Course. This course has been evaluated in a cluster RCT in 10 Australian schools [19,20] and was found to significantly improve alcohol and cannabis knowledge, reduce average alcohol consumption and the frequency of drinking to excess and using cannabis. These results, as well as positive feedback from students and teachers [19,21], provided the impetus to assess if this innovative delivery platform could be extended to other illicit drugs of concern.

Given that cannabis, ecstasy and amphetamines are among the most commonly used illicit drugs by Australian teenagers [2], as well as the huge potential for harm associated with their use [1,22,23], it was logical to develop a universal prevention program specifically targeting these drugs. As such, the six-lesson Climate Schools: Psychostimulant and Cannabis Module was developed for Year 10 students (aged 15–16 years), in consultation with students, teachers and health professionals. The current study sought to evaluate whether the Climate Schools: Psychostimulant and Cannabis Module was more effective than health education as usual in:

1) Increasing cannabis and psychostimulant-related knowledge

2) Reducing pro-drug attitudes about cannabis and psychostimulant use

3) Reducing the use of cannabis and psychostimulants

4) Reducing intentions to use psychostimulants and cannabis

## Methods

### Design

A cluster RCT, with schools as the unit of randomisation, was conducted with Year 10 students from 21 Independent and Catholic secondary schools in Sydney. Schools were randomly allocated, using the online tool *Research Randomiser*, to receive either the Climate Schools: Psychostimulant and Cannabis Module (intervention) or their usual Personal Development, Health and Physical Education (PDHPE) classes, including drug education, over the year (control). Students in both the intervention and control groups completed a self-report survey on four separate occasions; at baseline, immediately post-intervention, and at a five- and 10-month follow-up. Ethics approval was obtained from the University of New South Wales Human Research Ethics Committee (HREC 06252) and the trial was registered with the Australian and New Zealand Clinical Trials Registry (ACTRN12613000492752).

### Participants

Twenty-one schools with existing relationships with the researchers agreed to participate in the study. Written informed consent was required from students, their parent/guardians and teachers to participate in the study. Eleven schools were randomly allocated to the Climate Schools intervention group and ten schools were assigned to the control group. Written informed consent was provided by parents of 1839 Year 10 students, and students were required to provide informed consent themselves to participate. The final sample consisted of 1734 students (n = 906 Climate Schools intervention; n = 828 Control) at baseline. The sample had a mean age of 15.44 years (SD = 0.41) and 66.2% were male.

### Intervention

The Climate Schools: Psychostimulant and Cannabis Module is a six-lesson program aimed at decreasing cannabis and psychostimulant use and related harms. Each lesson is approximately 40-minutes long and consists of a 20-minute computer component, completed individually by students, followed by 20-minutes of teacher-delivered class activities. Consistent with the theoretical underpinnings of the existing Climate Schools modules, the program content was based on a social influence approach [24]. Full details of the topics covered in each lesson are listed in Table 1. All teachers were given a Teacher Manual which provided guidelines for implementing the program, but no formal training was given. The manual also included teacher and student summaries and five pre-planned classroom activities per lesson. Teachers were able to choose which activities to implement to ensure the activity suited the needs of their class.

**Table 1:** Lesson content of the Climate Schools: Psychostimulant and Cannabis Module

**Lesson**	**Content**
**1**	Cannabis: What is it?
	Short-term effects of cannabis
	Reasons people use cannabis
	Cannabis and the law
	Risk and protective factors for drug use
	Conservative norms: prevalence of cannabis use
	Mental health and cannabis
**2**	Critical analysis of drug-related internet and media resources
	Classifying drugs: hallucinogens, stimulants and depressants
	Psychostimulants: What are they?
	Common names and properties of psychostimulant drugs
	Short-term effects of psychostimulants
	Conservative norms: Prevalence of psychostimulant use
	The multifaceted nature of the effects of drug use on people’s lives
**3**	Definitions, examples and effects of poly-drug use
	Classifying drugs
	The indirect negative consequences of drug use
	Problem solving and decision making skills in relation to drugs
	Identifying drug related risk and minimising drug related harms
**4**	Communication skills
	Avenues for seeking help and barriers to accessing services
	What to do in a drug related emergency
	Calling 000, the emergency number
	Identifying communication styles, including assertiveness
**5**	Long-term effects of drugs
	Drug withdrawal
	Harm-minimisation strategies
	Learning about resilience
	Attitudes to drug use
	Prevalence of psychostimulants and cannabis use
	CPR and first aid
**6**	Drugs and driving
	Drugs and the law
	Problem solving skills
	Legalisation, decriminalisation and criminalization of drugs – the debate
	The effects of drugs on life’s journey
	The effects of drugs on others

### Measures

All students completed a 40-minute self-report questionnaire at baseline, immediately post-intervention, and at a 5- and 10-month follow-up. An overview of the intervention and assessment times is presented in Table 2. Student data was linked across time using a unique identification code based on easily remembered fragments of personal information, adapted from the *School Health and Alcohol Harm Reduction Project* (SHAHRP) [25]. Research staff administering the survey followed a standardised protocol and emphasised the confidential nature of the survey to students to encourage honest responding.

**Table 2:** Intervention and assessment times

	**Baseline**	**Intervention**	**Immediate post-intervention**	**5 month follow-up**	**10 month follow-up**
**Timing**	Term 2	Term 2&3	Term 2&3	Term 4	Term 2
	March-April	April-June	June-August	October-November	March-June
	2008	2008	2008	2008	2009
**Intervention group**	Survey 1	Climate Schools Intervention	Survey 2	Survey 3	Survey 4
**Control group**	Survey 1		Survey 2	Survey 3	Survey 4

#### Demographics

Demographic data included the respondents’ gender, age, average grades and number of days absent from school without parental permission in the previous school year.

#### Knowledge about cannabis and psychostimulants

Cannabis- and psychostimulant-related knowledge were each measured by a 15-item survey assessing knowledge about the prevalence of use, physical and mental health effects and social and legal consequences of these drugs. For each of item, students were required to indicate whether the statement was ‘true’, ‘false’ or whether they ‘don’t know’.

#### Attitudes to cannabis and psychostimulant use

Attitudes to cannabis and psychostimulant use were assessed using an adapted version of the Life Skills Training Questionnaire (LSTQ) [26]. Four items were used to assess attitudes to cannabis use, such as the perceived social benefits of using cannabis, with responses made on a five-point Likert scale, anchored by 1 (‘strongly disagree’) to 5 (‘strongly agree’). Attitudes to psychostimulants were measured using the same four items with ‘psychostimulants’ inserted in place of ‘cannabis’. The LSTQ attitude scale has acceptable internal consistency (α = 0.86).

#### Cannabis and psychostimulant use

Participants were asked to indicate if they had ever used cannabis or psychostimulants (meth/amphetamine and ecstasy), as well as any use in the past week, month, three and 12-months. Frequency of use was also assessed using a five point scale ranging from 1 (’every day’) to 5 (‘once or twice a year’). These questions were based on items from the National Drug Strategy Household Survey (NDSHS) 2004 [27] to allow for comparison with a large-scale representative group of Australians.

#### Intention to use drugs in the future

Six questions were used to assess students’ intentions to use cannabis, meth/amphetamine and ecstasy in the ‘next 12 months’ and in the ‘future’. Each question required students to rate their intention on a five point Likert scale ranging from 1 (‘very unlikely’) to 5 (‘very likely’).

#### Program evaluation and implementation

Students were asked to rate 10 statements regarding the quality, acceptability and likeability of the Climate Schools program. Intervention teachers were asked to complete a 14-item questionnaire rating the educational quality of the Climate Schools program, ease of access and acceptability of using a computer resource, accordance with curriculum content and intentions regarding future use of the program. Teachers were also asked to complete an implementation logbook at the completion of the program to indicate which optional activities they completed with their class and to comment on any factors that may have impacted the delivery of the intervention.

#### Control group questionnaire

Teachers at control schools were asked to complete a questionnaire to provide details on the timing and content of any psychostimulant and cannabis education they delivered to their Year 10 students over the course of the school year.

### Statistical methods

Attrition and differential attrition between groups were examined utilising the software program SPSS through a series of Analysis of Variance (ANOVA) for normally distributed data, Chi-squared and logistic regression for binomial and categorical data and Mann–Whitney U and Kruskal-Wallis for non-normally distributed data. To assess the effectiveness of the intervention, analyses were conducted on an Intention-to-Treat basis, that is, all students with baseline data were included in the analysis irrespective of the number of program lessons they completed. The HLM procedure used for the analyses accounted for missing data. This approach makes estimations based on available data points, meaning there was no need to remove students who have incomplete survey data at a given time point.

To determine the level of variability between schools, intraclass correlations (ICCs) were assessed on all outcomes. To account for clustering at the school level, hierarchical linear modelling (HLM) utilising HLM 6 [28,29] was conducted for normally distributed data (psychostimulant knowledge) while hierarchical generalised linear modelling (HGLM) using Poisson sampling was used for count data (lifetime use of cannabis and meth/amphetamine, frequency of cannabis, ecstasy and meth/amphetamine use and intention to use cannabis). All outcome variables were centred at post-test, allowing comparison between groups immediately after the completion of the intervention. For each outcome, intervention effects were explored in models which utilized linear and quadratic growth terms to characterise the pattern of change on the outcome over time. As recommended by Lee [30], HLM / HGLM procedures were abandoned in favour of single-level analyses (ANCOVA, logistic regression and hierarchical regression utilising SPSS) when the unconditional hierarchical model revealed that less than 10% of systematic variance existed at the between-school level. The outcome variables with ICCs below 10% were cannabis knowledge, cannabis and psychostimulant attitudes, lifetime use of ecstasy and intention to use meth/amphetamine and ecstasy in the next 12 months. For each outcome variable, to minimise the effects of loss to follow-up, three separate analyses were conducted to assess if there was a significant difference between the groups from baseline to each follow-up occasion. Bonferroni adjustments were made for multiple comparisons to control the type 1 error rate. That is, to adjust for the fact that separate analyses were conducted for baseline to immediate post-test, baseline to the 5-month follow-up and baseline to 10-month follow-up. Corresponding baseline scores were entered as a covariate into the models to account for differences between the intervention and control groups on outcomes at baseline. Although there is some evidence of converging rates of cannabis use between young males and females [31], considerable evidence suggests that males are more likely to use drugs than females. For this reason, in all analyses directly examining drug use behaviour, gender was also taken into account.

#### Sample size calculations

To account for cluster randomisation, sample size calculations were based on recent sample size requirements developed by Heo & Leon [32] to detect intervention by time interactions in longitudinal cluster randomized clinical trials. To allow for comparisons between groups, six schools in each group was required. This would achieve 80% power to detect a standardized between-group mean difference of 0.15 (p = 0.05) in use and knowledge outcomes at the end of the trial with three measurement occasions. An effect size of 0.15 is comparable to previous trials of universal drug prevention programs [33] and would have substantial benefits on a population level based on recent economic modelling [34].

## Results

### Sample

**Figure 1:**
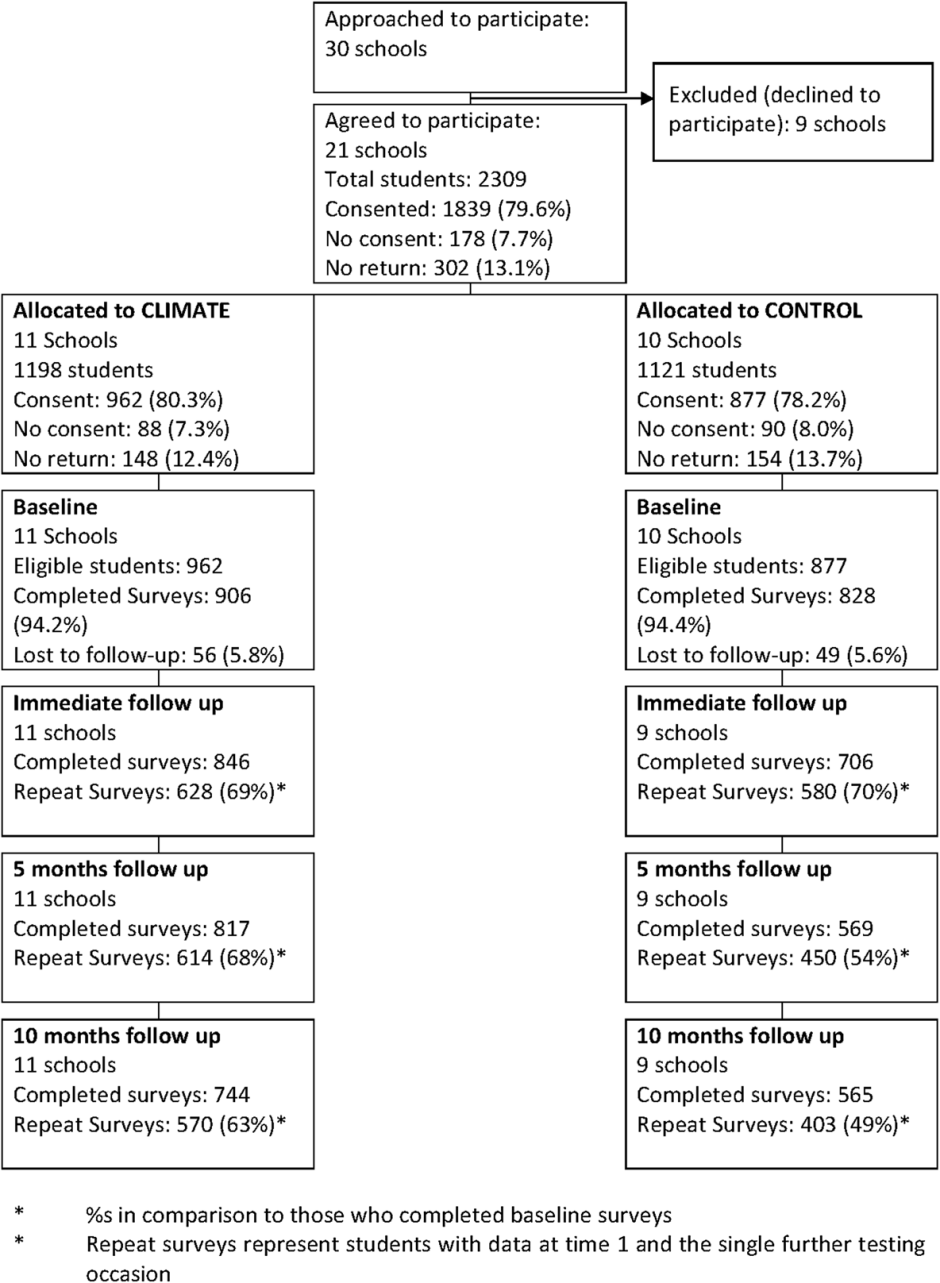
Consort flow diagram of recruitment and participation of schools.

A total of 1734 Year 10 students from 21 Independent and Catholic High Schools completed the survey at baseline (mean age = 15.44 years, SD = 0.41; 66.2% male). Of the baseline sample, 69.7% completed the survey immediately post-test, 61.4% provided data at five month follow-up and 56.1% were present at the 10-month follow-up. A total of 651 students (37.5%) had data for all four survey occasions. The number and percentage of students over time is presented in Figure 1.

### Baseline equivalence

Despite randomisation, at baseline, students in the intervention and the control groups differed on a number of outcome variables. Compared to the intervention group, the control group had greater pro-drug attitudes about psychostimulants (F(1, 1727) = 8.20, p < 0.01), and cannabis (F(1,1730) = 22.45, p < 0.001), greater lifetime cannabis use (χ2(1) = 18.47, p < 0.001), greater frequency of cannabis use (F(1, 1720) = 9.57, p = 0.002) and had greater intention to use cannabis (F(1,1729) = 21.77, p < 0.0001), meth/amphetamine F(1, 1728) = 9.48, p = 0.002 and ecstasy F(1,1727) = 5.77, p = 0.02 in the next 12 months.

### Attrition and differential attrition analyses

Attrition analyses were conducted to assess the comparability of those students who were present at baseline only (single), to those who were present at baseline and at least one further survey occasion (repeat) on outcomes measures. Table 3 indicates that students present only at baseline reported significantly higher levels of cannabis and psychostimulant use, greater intentions to use these substances and greater pro-drug attitudes. Attrition resulted from a number of different factors including one school withdrawing after the baseline survey due to changes in staff, time constraints prohibiting certain classes from completing the post-intervention survey, students being absent on the day of the survey, students no longer attending the school at the time of the survey, students failing to maintain the same identifier code, and students whose surveys were excluded as they had failed to include unique identifier codes or had not answered the questions.

**Table 3:** Differences between students who were retained beyond baseline (repeat) and those that were lost to follow-up (single) on baseline scores

**Outcome variable**	**Single**	**Repeat**	***df***	***df error***	***F***	***p***
Cannabis Knowledge	8.58	8.78	1	1727	1.46	p = 0.23
Psychostimulant Knowledge	8.26	8.51	1	1721	2.16	p = 0.14
Cannabis Attitudes	9.64	8.24	1	1730	47.24	p < 0.0001
Psychostimulant Attitudes	10.20	8.91	1	1730	41.57	p < 0.0001
Cannabis frequency of use (three months)	0.47	0.12	1	1720	48.25	p < 0.001
Meth/amphetamine frequency of use (12 months)	0.22	0.07	1	1727	17.61	p < 0.001
Ecstasy frequency of use (last 12 months)	0.29	0.09	1	1727	29.8	p < 0.001
Cannabis intention 12 months	1.10	0.60	1	1729	44.61	p < 0.001
Meth/amphetamine intention 12 months	0.62	0.30	1	1727	38.20	p < 0.001
Ecstasy intention 12 months	0.71	0.38	1	1727	29.66	p < 0.001
	**Single**	**Repeat**	**df**		**χ**^***2***^	***p***
Lifetime cannabis use	0.22	0.10	1		33.58	p < 0.0001
Lifetime Methamphetamine use	0.10	0.03	1		27.37	p < 0.0001
Lifetime ecstasy use	0.12	0.05	1		32.2	p < 0.0001

Differential attrition analyses were conducted to assess if there were any differences between the intervention and control condition in the students who were retained beyond baseline (repeat) and those that were lost to follow-up (single). On all outcome variables, there was no evidence of differential attrition.

### Intervention effects

Tables 4, 5, 6 and 7 provide the descriptive statistics for all outcome variables over the four survey occasions for the intervention and the control groups.

**Table 4:** Mean knowledge and attitude scores (95% confidence intervals and sample size) for the intervention and control groups over time

		**Baseline**	**Post**	**Five month follow-up**	**10-month follow-up**
**Cannabis Knowledge**^**a**^	**Control**	8.81	9.25	9.14	9.51
		(8.63-8.98)	(9.04-9.46)	(8.87-9.41)	(9.24-9.78)
		(n = 826)	(n = 576)	(n = 448)	(n = 403)
	**Intervention**	8.69	10.76	10.42	10.39
		(8.52-8.86)	(10.58-10.94)	(10.22-10.62)	(10.17-10.61)
		(n = 902)	(n = 627)	(n = 612)	(n = 570)
**Psycho-stimulants knowledge**^**a**^	**Control**	8.49	9.08	8.89	9.34
		(8.31-8.67)	(8.86-9.30)	(8.60-9.18)	(9.05-9.63)
		(n = 823)	(n = 577)	(n = 449)	(n = 403)
	**Intervention**	8.45	10.46	10.25	10.28
		(8.27-8.63)	(10.26-10.66)	(10.03-10.47)	(10.04-10.52)
		(n = 899)	(n = 627)	(n = 613)	(n = 569)
**Cannabis Attitude**^**b**^	**Control**	8.86	8.71	8.61	8.85
		(8.62-9.10)	(8.46-8.96)	(8.32-8.90)	(8.54-9.16)
		(n = 826)	(n = 578)	(n = 445)	(n = 403)
	**Intervention**	8.13	8.04	8.30	7.95
		(7.93-8.33)	(7.80-8.28)	(8.05-8.55)	(7.70-8.20)
		(n = 905)	(n = 626)	(n = 612)	(n = 568)
**Psycho-stimulants Attitude**^**b**^	**Control**	9.36	9.55	9.34	9.68
		(9.14-9.58)	(9.30-9.80)	(9.05-9.63)	(9.37-9.99)
		(n = 824)	(n = 577)	(n = 445)	(n = 403)
	**Intervention**	8.93	8.88	8.96	8.74
		(8.71-9.15)	(8.63-9.13)	(8.71-9.21)	(8.49-8.99)
		(n = 904)	(n = 626)	(n = 614)	(n = 568)

^a^Knowledge scores can range from 0–15; with a higher score reflecting greater knowledge.

^b^Attitude scores range from 4–20; with a higher score reflecting a more positive attitude towards the use of drugs.

**Table 5:** The proportion of students who reported having ever used cannabis, meth/amphetamine or ecstasy in their lifetime for the intervention and control groups over time (95% confidence intervals and sample size)

		**Baseline**	**Post-intervention**	**Five month follow-up**	**10 month follow-up**
**Proportion ever used cannabis**	**Control**	0.15	0.16	0.16	0.20
		(0.13-0.17)	(0.13-0.19)	(0.13-0.19)	(0.16-0.24)
		(n = 828)	(n = 580)	(n = 450)	(n = 403)
	**Intervention**	0.09	0.11	0.13	0.12
		(0.07-0.11)	(0.09-0.13)	(0.10-0.16)	(0.09-0.15)
		(n = 903)	(n = 627)	(n = 614)	(n = 570)
**Proportion ever used meth/amphetamine**	**Control**	0.05	0.06	0.07	0.05
		(0.04-0.06)	(0.04-0.08)	(0.05-0.09)	(0.03-0.07)
		(n = 827)	(n = 577)	(n =449)	(n = 403)
	**Intervention**	0.04	0.03	0.05	0.04
		(0.03-0.05)	(0.02-0.04)	(0.03-0.07)	(0.02-0.06)
		(n = 906)	(n = 627	(n = 613)	(n = 570)
**Proportion ever used ecstasy**	**Control**	0.07	0.09	0.09	0.07
		(0.05-0.09)	(0.07-0.11)	(0.07-0.12)	(0.05-0.09)
		(n = 827)	(n = 580)	(n = 449)	(n = 403)
	**Intervention**	0.05	0.05	0.06	0.06
		(0.04-0.07)	(0.03-0.06)	(0.04-0.08)	(0.04-0.08)
		(n = 904)	(n = 627)	(n = 614)	(n = 569)

**Table 6:** Mean frequency of cannabis, meth/amphetamine and ecstasy use (95% confidence interval and sample size), for the intervention and control groups, over time

		**Baseline**	**Post**	**Five month follow-up**	**10 month follow-up**
**Frequency of cannabis use in last three months**^**a**^	**Control**	0.24	0.25	0.27	0.29
		(0.18-0.30)	(0.17-0.33)	(0.19-0.35)	(0.19-0.39)
		(n = 823)	(n = 579)	(n = 447)	(n = 403)
	**Intervention**	0.12	0.15	0.22	0.16
		(0.08-0.16)	(0.09-0.21)	(0.14-0.30)	(0.10-0.22)
		(n = 898)	(n = 626)	(n = 614)	(n = 568)
**Frequency of meth/amphetamine use in last 12 months**^**b**^	**Control**	0.12	0.13	0.09	0.10
		(0.04-0.12)	(0.02-0.10)	(0.07-0.15)	(0.07-0.15)
		(n = 824)	(n = 578)	(n = 449)	(n = 403)
	**Intervention**	0.08	0.06	0.11	0.09
		(0.04-0.12)	(0.02-0.10)	(0.07-0.15)	(0.05-0.13)
		(n = 904)	(n = 628)	(n = 614)	(n = 569)
**Frequency of ecstasy use in last 12 months**^**b**^	**Control**	0.15	0.18	0.16	0.12
		(0.11-0.19)	(0.12-0.24)	(0.10-0.22)	(0.10-0.22)
		(n = 824)	(n = 578)	(n = 447)	(n = 403)
	**Intervention**	0.10	0.10	0.12	0.11
		(0.06-0.14)	(0.06-0.14)	(0.08-0.16)	(0.07-0.15)
		(n = 904)	(n = 628)	(n = 614)	(n = 569)

^a^Frequency was coded as: 0 = Have not used in the last 3 months, 1 = About once a month, 2 = Less than once a week, 3 = About once a week, 4 = More than once a week (but less than daily), 5 = Once a day, 6 = More than once per day.

^b^Frequency was coded as: 0 = Have not used in the last 12 months, 1 = once or twice a year, 2 = Every few months, 3 = About once a month, 4 = Once a week or more, 5 = every day.

**Table 7:** Mean (95% confidence interval and sample size) likelihood ratings for using cannabis, methamphetamine or ecstasy in the next 12 months for the intervention and control groups on each survey occasion

		**Baseline**	**Post**	**Five month follow-up**	**10 month follow-up**
**Intention to use cannabis in the next 12 months**	**Control**	0.82	0.92	0.93	0.95
		(0.74-0.90)	(0.80-1.04)	(0.79-1.07)	(0.81-1.09)
		(n = 827)	(n = 579)	(n = 448)	(n = 403)
	**Intervention**	0.56	0.62	0.72	0.61
		(0.48-0.64)	(0.54-0.70)	(0.62-0.82)	(0.51-0.71)
		(n = 903)	N = 627	(n = 614)	(n = 570)
**Intention to use methamphetamine in the next 12 months**	**Control**	0.41	0.52	0.45	0.49
		(0.35-0.47)	(0.44-0.60)	(0.35-0.55)	(0.39-0.59)
		(n = 827)	(n = 577)	(n = 447)	(n = 403)
	**Intervention**	0.29	0.34	0.36	0.36
		(0.23-0.35)	(0.28-0.40)	(0.28-0.44)	(0.28-0.44)
		(n = 902)	(n = 625)	(n = 614)	(n = 569)
**Intention to use ecstasy in the next 12 months**	**Control**	0.50	0.63	0.63	0.62
		(0.44-0.56)	(0.53-0.73)	(0.53-0.73)	(0.50-0.74)
		(n = 823)	(n = 577)	(n = 445)	(n = 402)
	**Intervention**	0.39	0.40	0.49	0.46
		(0.33-0.45)	(0.32-0.48)	(0.40-0.56)	(0.38-0.54)
		(n = 905)	(n = 625)	(n = 614)	(n = 569)

#### Cannabis-related knowledge

Immediately post intervention there was a significant difference between the intervention and control groups (F(1,1196) = 178.74, p < 0.001), with students in the intervention group reporting significantly higher levels of cannabis-related knowledge than those in the control group. The intervention group continued to have significantly higher levels of knowledge than the control group (F(1,1054) = 73.31, p < 0.001) five months post intervention, and at the 10 month follow-up (F(1,970) = 34.01, p < 0.001).

#### Psychostimulant-related knowledge

The unconditional linear model revealed that 12.5% of the variance could be accounted for at the between-school level. Intervention effects were explored in a model which utilised a linear and quadratic growth term to characterise the pattern of change in knowledge scores over time. The inclusion of intervention effects significantly improved model fit (χ^2^(3) = 21.12, p < 0.0001) and explained 59% of the variance in post-test knowledge scores and 68% and 71% of the variance in linear and quadratic growth respectively. According to this model, the population mean knowledge score at post-test was 8.82, and there was a significant effect of group, with students in the intervention group answering 1.33 more items correctly on the knowledge questionnaire than students in the control group. There was significant average population growth in knowledge of 0.30 items per survey occasion. There was also a significant group effect, indicating that for students in the intervention group, their knowledge score increased by an extra 0.78 items per survey occasion compared with the control group. However, growth in scores slowed down by 0.47 items per occasion for the intervention group only. This suggests that although there was a significant difference between groups, this difference diminished with time.

#### Attitudes to cannabis use

Immediately post intervention there was no significant difference between the intervention and control group (F(1,1201) = 3.76, p = 0.053) in cannabis related attitudes. At five-months post intervention, the intervention and control groups still did not differ significantly in their attitudes towards cannabis use (F(1,1056) = 1.46, p = 0.637). By 10 months post intervention, however, the intervention group had significantly lower pro-cannabis attitudes than the control group (F(1,970) = 7.16, p = 0.008).

#### Attitudes to psychostimulant use

Immediately post-intervention there was a significant difference between groups (F(1,1200) = 6.98, p = 0.008), with students in the control group having significantly higher pro-psychostimulant attitudes than students in the intervention group, even after adjusting for baseline differences. Although this difference was no longer significant five months post-intervention (F(1,1057) = 4.05, p = 0.045), by the 10-month follow-up the intervention group once again had significantly lower pro-psychostimulant attitudes than the control group (F(1,967) = 15.02, p < 0.001).

#### Ever used cannabis

The unconditional linear model revealed that 18.70% of the variance could be accounted for at the between-school level. Intervention effects were explored in a model which utilised a linear growth term to characterise the pattern of change in use over time. Gender was added to the model and found to be a significant predictor of the proportion of students who reported having ever used cannabis at post-test (OR = 0.52, 95% CI: 0.32-0.85), with females being significantly less likely to report having ever used. Gender was not a significant predictor of the linear growth in the odds of having used cannabis over time (OR = 1.00, 95% CI: 0.84-1.18). Intervention was not found to be a significant predictor of post-test scores (OR = 0.67, 95% CI: 0.31-1.44) or linear growth over time (OR = 1.05, 95% CI: 0.92-1.20).

#### Ever used meth/amphetamine

The unconditional linear model revealed that 16.54% of the variance could be accounted for at the between-school level. Intervention effects were explored in a model which utilised a linear growth term to characterise the pattern of change in use over time. Gender was added to the model and was found to be a significant predictor of the proportion of students who reported having ever used meth/amphetamine at post-test (OR = 0.65, 95% CI: 0.42-0.99), with girls being significantly less likely to report having ever used. Gender was not a significant predictor of the linear growth in the odds of having used methamphetamine over time (OR = 1.05, 95% CI: 0.86-1.28). Intervention was not found to be a significant predictor of post-test scores (OR = 0.60, 95% CI: 0.29-1.26) or linear growth over time (OR = 1.02, 95% CI: 0.85-1.22).

#### Ever used ecstasy

Lifetime ecstasy use at baseline and gender were entered as covariates in the model. At post-test, gender was not a significant predictor of lifetime ecstasy use (OR: 1.56, 95% CI: 0.83-2.92), but intervention condition was a significant predictor. The results revealed that students in the control group were twice as likely to have ever used ecstasy in their lifetime compared with students in the intervention group (OR: 2.27 95% CI: 1.20-4.27). At neither the five-month (OR: 1.30 95% CI: 0.74-2.28) nor 10-month (OR: 1.83 95% CI: 0.97-3.43) follow-up was gender a significant predictor of having ever used ecstasy. At both the five-month (OR: 1.56 95% CI: 0.91-2.68) and 10-month (OR: 1.00 95% CI: 0.55-1.81) follow-up, intervention condition was also not a significant predictor of having ever used ecstasy after taking baseline and gender into account.

#### Frequency of cannabis use in the last three months

The unconditional linear model revealed that 20.57% of the variance could be accounted for at the between-school level. Intervention effects were explored in a model which utilised a linear and quadratic growth term to characterise the pattern of change in use over time. Gender was added to the model and significantly predicted post-test scores and quadratic growth in the frequency of cannabis use in the last three months. Specifically, at post-test, the frequency of use for females was significantly lower than for males (Event rate ratio = 0.54, 95% 0.34-0.85), but the quadratic growth was greater for females than males over time (Event rate ratio = 1.21, 95% CI: 1.02-1.43). Gender was not a significant predictor of linear growth in the frequency of last three month cannabis use over time (Event rate ratio = 1.12, 95% CI: 0.86-1.45) and hence was removed as a predictor of linear growth over time. Intervention was not a significant predictor of the frequency of last three month cannabis use at post-test (Event rate ratio = 0.65, 95% CI: 0.29-1.47) or linear (Event rate ratio = 1.15, 95% CI: 0.97-1.39) and quadratic (Event rate ratio = 1.03, 95% CI: 0.93-1.13) growth over time. Intervention did, however, interact with gender in predicting quadratic growth over time. Specifically, females in the intervention group had a significantly greater decline in quadratic growth of cannabis use over time (Even rate ratio: 0.60, 95% CI: 0.51-0.71).

#### Frequency of meth/amphetamine in the last 12 months

The unconditional linear model revealed that 11.54% of the variance could be accounted for at the between-school level. Intervention effects were explored in a model which utilised a linear growth term to characterise the pattern of change in use over time. Gender was added as a school level predictor to the model and significantly predicted post-test scores. Specifically, at post-test, the frequency of use for females was significantly lower than for males (Event rate ratio = 0.54, 95% 0.34-0.86). Gender was not a significant predictor of linear growth in the frequency of last 12 month methamphetamine use over time (Event rate ratio = 0.84, 95% CI: 0.67-1.05) and, hence, was removed as a predictor of linear growth over time. Intervention was not a significant predictor of the frequency of last 12 month methamphetamine use at post-test (Event rate ratio = 0.71, 95% CI: 0.37-1.39) or linear growth over time (Event rate ratio = 1.17, 95% CI: 0.92-1.49). There was also no evidence of gender by group interaction on post-test scores (Event rate ratio = 1.08, 95% CI: 0.43-2.69).

#### Frequency of ecstasy use last 12 months

The unconditional linear model revealed that 10.52% of the variance could be accounted for at the between-school level. Intervention effects were explored in a model which utilised a linear and quadratic growth term to characterise the pattern of change in use over time. Gender was added to the model and significantly predicted post-test scores. Specifically, at post-test, the frequency of use for females was significantly lower than for males (Event rate ratio = 0.61, 95% 0.34-0.99). Gender was not a significant predictor of linear (Event rate ratio = 0.97, 95% CI: 0.75-1.26) or quadratic growth (Event rate ratio = 0.91, 95% CI: 0.77-1.07) in the frequency of last 12 month ecstasy use over time and hence was removed as a predictor of linear and quadratic growth over time. Intervention was found to be a significant predictor of the frequency of last 12 month ecstasy use at post-test (Event rate ratio = 0.50, 95% CI: 0.26-0.95), specifically, the intervention group demonstrated a significantly lower frequency of ecstasy use in comparison with the control. By contrast, the intervention group did demonstrate significantly greater quadratic growth in the frequency of ecstasy use over time (Event rate ratio = 1.17, 95% CI: 1.04-1.31). There was no significant difference between groups in the rate of linear growth in the frequency of ecstasy use over time (Event rate ratio = 0.95, 95% CI: 0.82-1.12).

#### Intention to use cannabis in the next 12 months

The unconditional linear model revealed that 10.17% of the variance could be accounted for at the between-school level. Intervention effects were explored in a model which utilised a linear and quadratic growth term to characterise the pattern of change in use over time. Intervention was not found to be a significant predictor of the intention to use cannabis in the next 12 months (Event rate ratio = 0.71, 95% CI: 0.47-1.08), linear (Event rate ratio = 1.00, 95% CI: 0.93-1.07) or quadratic growth (Event rate ratio = 1.01, 95% CI: 0.96-1.07) over time.

#### Intention to use meth/amphetamine in the next 12 months

Immediately post-intervention there was a significant difference between the intervention and control groups (F(1,1200) = 5.96, p = 0.01). Students in the control group had significantly greater intention to use methamphetamine in the next 12 months, however, for both groups, intention to use was very low. At both five months (F(1,1059) = 1.15, p = 0.28) and 10 months (F(1,972) = 1.14, p = 0.29) post-intervention, there were no significant differences between the groups in their reported intention to use methamphetamine in the next 12 months, with both groups reporting minimal intention.

#### Intention to use ecstasy in the next 12 months

Immediately post-intervention there was a significant difference between the intervention and control groups (F (1,1199) = 10.64, p = 0.001) with students in the control group reporting significantly greater intention to use ecstasy in the next 12 months in comparison to the intervention group. A trend in this direction remained at the five-month follow-up (F(1,1055) = 5.21, p = 0.02), however by the 10-month follow-up there was no a significant difference between groups (F(1, 970) = 1.04, p = 0.31). On all occasions, the intention of students in both groups to use ecstasy in the next 12 months was minimal.

### Program evaluation

A total of 749 students and 34 teachers completed an evaluation survey about the Climate Schools course. Feedback indicated that the program was well received and enjoyed by both teachers and students. The majority of students (71%) indicated that the cartoon story was an enjoyable way of learning and 63% said that they would like to learn other health theory topics in this way. Eighty-seven per cent of teachers reported that students could recall the information taught in the computer component ‘very well’ or ‘well’. In terms of implementing a computer-based program, the vast majority of teachers reported that it was ‘very easy’ (45%) or ‘easy’ (42%) to implement. Although most teachers did not have problems accessing computer resources in their schools, some teachers (23%) did report difficulties. Ninety-seven per cent of teachers said that they would be likely to use the program in the future and 94% reported that they would be likely to recommend it to other teachers. Teachers varied widely in which classroom activities, and how many activities, they completed with their class. Over eighty per cent of teachers rated the educational quality of the activities positively and thought the activities were helpful in reinforcing information about psychostimulants and cannabis to students.

### Control school cannabis and psychostimulant education

Eight schools in the control condition provided details of the content and timing of the cannabis and psychostimulant education that was provided to their students. Three schools delivered a social influence program based on a harm-minimisation approach, one school delivered a harm-minimisation program and four schools did not cover psychostimulants as a topic at all. The number of lessons spent on cannabis and psychostimulant education varied, ranging from eight to 13.5 lessons.

## Discussion

### Summary of findings

The Climate Schools program was shown to increase cannabis and psychostimulant-related knowledge and decrease pro-drug attitudes. Immediately after the intervention, students who received the Climate Schools program had significantly higher levels of knowledge than the control group. The absolute difference in knowledge between groups did diminish over time, but was still significantly different 10 months post-intervention. The capacity to positively impact on knowledge and attitudes is consistent with a large body of previous research on school-based drug prevention [35-38].

These results directly challenge the notion that harm-minimisation information is too complex for young people to learn, a justification used to support the teaching of simpler abstinence based messages alone [39]. Specifically, consistent with a harm-minimisation approach, the knowledge taught in the current module was to strongly encourage young people to refrain from drug use, but it also provided the knowledge required to practice harm-minimisation skills to prevent harms as a result of one’s own or other people’s drug use. The demonstrated increase in knowledge in the intervention group provides evidence that young people can learn harm-minimisation information. These findings are consistent with previous Climate Schools research [18-21] and the SHAHRP program which was also conducted in Australia [40]. The heartening aspect of the results from the current study is that students in both the intervention and control groups predominantly had very negative attitudes towards drug use from the outset of this study, attitudes which became even more negative in the intervention group after they received the intervention.

Although it is important to change young people’s attitudes to drug use, the research has clearly demonstrated that this is not sufficient to achieve behavioural change [41] and the demonstration of behavioural change is what is considered to be essential [36,42,43]. The behavioural change of interest in this study is to alter the otherwise predicted course of development, which is either to suppress drug use behaviour or keep it from occurring [44].

The Climate Schools program was effective in delaying initiation to ecstasy use for the intervention group, compared to the control group. Even though this difference between groups diminished by the five-month follow-up, it is an important result as a substantial body of evidence has shown that early onset of drug use is a risk factor for developing a substance use disorder in later life [45,46]. The Climate Schools program was also related to a plateau in the frequency of ecstasy use in the intervention group, with this difference gradually diminishing over time. Halting the use and frequency of ecstasy use is vital, because even though serious acute adverse events related to ecstasy may be relatively rare, when they do occur they are unpredictable and are associated with considerable morbidity and mortality [23]. Although a plateauing of frequency of ecstasy use was demonstrated in the current study, it is essential to also bear in mind that the overall prevalence of use in the current sample was very low and could at most be described as experimental use.

The Climate Schools program did not delay initiation to cannabis use or impact on cannabis related harms, but it was related to decreasing the frequency of cannabis use for females, although not for males. Although the frequency of cannabis use in the current sample is typical of occasional and experimental cannabis use, even the smallest reductions in frequency of use may assist in subduing the growth in trajectories to riskier levels of use in the future. The fact that the Climate Schools program was not successful in delaying initiation to cannabis use can likely be explained by research that indicates that for prevention to be effective in delaying initiation, it needs to be delivered early. That is, prevention for cannabis needs to be delivered before experimentation occurs and that by 15 years of age, it is too late to deliver a standalone intervention for the prevention of cannabis use, as has been done in the current trial. Research does, however, suggest that ongoing interventions are also important, as young people who cease cannabis use or decrease the frequency of use have better prognoses for the future [47]. This supports the need for sequential and developmentally appropriate interventions, which have now been catered for within the Climate Schools drug prevention resource.

The lack of preventive effects for males in changing the frequency of cannabis use is consistent with a growing body of evidence which shows that school-based prevention programs may be less effective for males than females [19,48]. Although there is some evidence of converging rates of cannabis use between young males and females [31,49], considerable evidence suggests that males are not only more likely to have tried cannabis, but are also more likely to become long-term problematic users [50]. The cannabis use of male students in this sample was also elevated in comparison with females, with males being significantly more likely to have ever used cannabis and use cannabis more frequently post-test. Males were significantly more likely to have ever used meth/amphetamine at post-test, in the last three months, and used more frequently in the last 12 months. The majority of these differences between males and females remained consistent over the duration of the study. Likewise for ecstasy, with the exception of lifetime use, males were more likely to have used ecstasy in the last three months and used more frequently in the last 12 months.

The propensity for males to be a higher risk group may be a result of the differences in the level of risk and protective factors for drug use experienced by males in comparison with females [51]. In particular, research suggests that the biological and social consequences of drug use may be stronger protective factors for females in comparison with males; in particular, greater social sanction against drug use by females in comparison with males [51,52]. Whereas for males, it may be higher levels of impulsivity or the need to use drugs with the motivation of enhancing their capacity to cope that explain some of the increased levels of drug use, more so than for females [53]. Gaining a greater understanding of the risk and protective factors which lead to gender differences in drug use is essential because a more comprehensive understanding of these factors could assist in making prevention program equally and more effective for both males and females.

The Climate Schools program was not effective in changing meth/amphetamine use. This result may explained by the low levels of use in the sample (4-5%) and the lack of variation in use both within and between schools over the study duration, which were evident in the HLM analyses. Students’ intentions to use cannabis in the next 12 months did not significantly differ among the intervention and control groups at any of the survey occasions. Although students in the control group reported significantly greater intent to use meth/amphetamine than the intervention group immediately post-intervention, this effect did not persist at the later follow-up occasions. Similarly, by the 10-month follow-up the intervention and control groups did not differ in terms of intent to use ecstasy in the next 12 months. These findings are likely to reflect the very low levels of intention reported by students in both groups to use cannabis, meth/amphetamine and ecstasy throughout the study period.

Finally, it is important to note that the Climate Schools program was well received by teachers and students. Feedback from the current study indicates that it was feasible to implement a six-lesson computer-based program about illicit substances to Year 10 students. By delivering the program via computers, traditional obstacles to successful implementation, such as the need for teacher training and high costs, appear to have been overcome. Although some teachers reported finding it difficult to access computers to deliver the program, there have been improvements in access to computer technology at Australian schools in the past six years since this trial was conducted and several recent initiatives. For example, the Bring Your Own Device (BYOD) scheme, in which students are encouraged to bring their own laptop or tablet to school, is likely to increase access to computers and improve the feasibility of using computer- and Internet-based programs.

### Limitations

Despite randomisation, the control group was typically a higher risk group than the intervention group at baseline on a number of drug use measures. Although all the analyses took baseline scores into account to control for differences between the intervention and control group, this does not necessarily control for the possibility that the control group may have a higher-risk trajectory over time.

In terms of the analyses employed in the current study, it should be noted that ANCOVA and hierarchical regression procedures are not necessarily the most powerful alternatives when assumptions violated. In recent years, Generalized Estimating Equations (GEEs) have emerged as a superior approach for analysing longitudinal repeated measures data [54,55] and as such, it would be beneficial to conduct future analyses using a GEE approach.

The attrition of high-risk students, which is a common occurrence in school-based prevention research [56-59], is also potentially problematic. Attrition of high-risk students has the potential to limit the external validity of findings and may also result in an overestimation of program effects. In the current study there was no evidence of differential attrition, which suggests that the prevention effects which have been detected are not spurious positive findings which have resulted from higher-risk subjects dropping out of the intervention group rather than the control condition.

Another limitation pertains to the accuracy of collecting self-report data on substance use. However, there is evidence to support the validity of self-report of alcohol and other drug use with adolescents [60-62]. In the current study, assurances of confidentiality were repeatedly provided during survey administration [63] and clear information was provided about the anonymity of individuals and schools in the provision of results [44], factors which have both been shown to enhance self-report accuracy.

Finally, the current study was powered for a standardized difference of 0.15, which is consistent with effects reported for other universal school-based interventions for alcohol and other drugs [33,64]. Such a modest size of effect is most impactful when applied across large populations and the Internet-based delivery of the Climate Schools program makes such large-scale implementation possible. However, a limitation of universal approaches is their limited impact on adolescents who are most highly at risk of developing problems or who have already developed problems. Targeted prevention programs specifically tailored to the needs of these individuals, such as the selective personality-targeted *Preventure* program [65,66], may be more suitable for high-risk students and produce greater effects.

## Conclusions

The Climate Schools: Psychostimulant and Cannabis Module was found to increase cannabis- and psychostimulant-related knowledge and was also effective in modifying pro-drug attitudes. Compared to drug education as usual, the computer-delivered harm-minimisation program led to a plateau in the frequency of ecstasy use as well as reducing the frequency of cannabis use (for females only). The Climate Schools program was successful in reducing intent to use ecstasy and meth/amphetamine in the short-term, however these effects did not persist over time. No changes were demonstrated for meth/amphetamine use behaviour. These findings, together with positive evaluations from teachers and students, provide further support that prevention programs based on a harm-minimisation approach and delivered by computer offer an innovative new platform for the delivery of prevention education for both licit and illicit drugs in schools.

## Competing interests

The authors declare that they have no competing interests.

## Authors’ contributions

LV & MT conceptualised and designed the study, and gained funding to carry it out. LV managed all aspects of the study including implementation of interventions into schools. LV & NN were directly involved in data collection and LV led the data analysis. All authors were involved in the write up of results and have read and approved the final manuscript.

## References

[B1] Australian Institute of Health and Welfare 2010 National Drug Strategy Household Survey report 2011 AIHW: Drug statistics series no 25 Cat no PHE 145 Canberra

[B2] White V Bariola E Australian secondary school students’ use of tobacco, alcohol, and over-the-counter and illicit substances in 2011. In 2012 Drug Strategy Branch, Australian Government Department of Health and Ageing.: Canberra

[B3] McLaren J Mattick RP Cannabis in Australia: Use, supply, harms, and response 2007 Australian Government Department of Health and Ageing: Prepared by National Drug and Alcohol Research Centre for the Drug Strategy Branch

[B4] Copeland J Swift W Cannabis use disorder: epidemiology and management Int Rev Psychiatry 2009 21 96 103 19367503 10.1080/09540260902782745

[B5] McLaren J Lemon J Robins L Mattick RP Cannabis and Mental Health: Put into Context 2008 Australian Government Department of Health and Ageing: Prepared by the National Drug and Alcohol Research Centre for the National Drug Strategy

[B6] Botvin GJ Preventing drug abuse in schools: Social and competence enhancement approaches targeting individual-level etiologic factors Addict Behav 2000 25 6 887 897 11125777 10.1016/s0306-4603(00)00119-2

[B7] Shin HS A review of school-based drug prevention program evaluations in the 1990's Am J Health Educ 2001 32 3 139 147

[B8] Wenter DL Ennett ST Ribisl KM Vincus AA Rohrbach L Ringwalt CL Jones SM Comprehensiveness of substance use prevention programs in US middle schools J Adolesc Health 2002 30 6 455 462 12039516 10.1016/s1054-139x(02)00346-4

[B9] Midford R McBride N Munro G Harm reduction in school drug education: Developing an Australian approach Drug Alcohol Rev 1998 17 3 319 327 16203498 10.1080/09595239800187151

[B10] Cuijpers P Three decades of drug prevention research Drugs: Education, Prevention & Policy 2003 10 1 7 20

[B11] Dusenbury L Brannigan R Falco M Hansen W A review of research on fidelity of implementation: implications for drug abuse prevention in school settings Health Educ Res Theory Pract 2003 18 2 237 256 10.1093/her/18.2.237 12729182

[B12] Kaftarian S Robinson E Compton W Watts Davis B Valkow N Blending prevention research and practice in schools: Critical issues and suggestions Prev Sci 2004 5 1 1 3 15058906 10.1023/b:prev.0000013975.74774.bc

[B13] Ringwalt C Ennett S Vincus A Thorne J Rohrbach LA Simons-Rudolph A The prevalence of effective substance use prevention curricula in U.S. middle schools Prev Sci 2002 3 4 257 265 12458764 10.1023/a:1020872424136

[B14] Rohrbach L Graham J Hansen W Diffusion of a school-based substance abuse prevention program: Predictors of program implementation Prev Med 1993 22 2 237 260 8483862 10.1006/pmed.1993.1020

[B15] Backer TE Finding the balance: Program fidelity and adaptation in substance abuse prevention: a state of the art review 2001 Rockville: In. Edited by Center for Substance Abuse Prevention SAaMHSA

[B16] Pankratz MM Jackson-Newsom J Giles SM Ringwalt CL Bliss K Bell ML Implementation fidelity in a teacher-led alcohol use prevention curriculum J Drug Educ 2006 36 4 317 333 17533804 10.2190/H210-2N47-5X5T-21U4

[B17] Vogl LE Teesson M Newton NC Andrews G Developing a school-based drug prevention program to overcome barriers to effective program implementation: The CLIMATE Schools: Alcohol Module Open J Prev Med 2012 2 3 410 422

[B18] Newton NC Vogl LE Teesson M Andrews G CLIMATE Schools: alcohol module: cross-validation of a school-based prevention programme for alcohol misuse Aust N Z J Psychiatry 2009 43 3 201 207 19221908 10.1080/00048670802653364

[B19] Vogl L Teesson M Andrews G Bird K Steadman B Dillon P A computerized harm minimization prevention program for alcohol misuse and related harms: randomized controlled trial Addiction 2009 104 4 564 575 19335655 10.1111/j.1360-0443.2009.02510.x

[B20] Newton NC Andrews G Teesson M Vogl LE Delivering prevention for alcohol and cannabis using the Internet: a cluster randomised controlled trial Prev Med 2009 48 6 579 584 19389420 10.1016/j.ypmed.2009.04.009

[B21] Newton NC Teesson M Vogl LE Andrews G Internet-based prevention for alcohol and cannabis use: final results of the Climate Schools course Addiction 2010 105 4 749 759 20148791 10.1111/j.1360-0443.2009.02853.x

[B22] Hall W Degenhardt L Adverse health effects of non-medical cannabis use Lancet 2009 374 1383 1391 19837255 10.1016/S0140-6736(09)61037-0

[B23] Gowing LR Henry-Edwards SM Irvine RJ The health effects of ecstasy: a literature review Drug Alcohol Rev 2002 21 53 63 12189005 10.1080/09595230220119363

[B24] Botvin GJ Preventing drug abuse in schools: social and competence enhancement approaches targeting individual-level etiologic factors Addict Behav 2000 25 6 887 897 11125777 10.1016/s0306-4603(00)00119-2

[B25] McBride N Farringdon F Muleners L Midford R School Health and Alcohol Harm Reduction Project: Details of intervention development and research procedures. In 2006 Perth, W.A.: National Drug Research Institute, Curtin University of Technology

[B26] National Health Promotion Association LifeSkills 2004 Life Skills Training Questionnaire - Instruction Guide

[B27] AIHW National Drug Strategy Household Survey 2005 Drug Statistics Series No 13. Canberra: AIHW: First results

[B28] Raudenbush S Bryk A Cheong YF Congdon R du Toit M HLM6 2004 Scientific Software International: Hierarchical Linear and Non-Linear Modeling. Lincolnwood: SSI

[B29] Raudenbush S Bryk AS Hierarchical Linear Models: Applications and Data Analysis Methods 2002 Newbury Park, CA: Sage

[B30] Lee VE Using hierarchical linear modeling to study social contexts: The case of school effects Educational Psychologist 2000 35 2 125 141

[B31] Degenhardt L Chiu W Sampson N Kessler RC Anthony JC Angermeyer M Bruffaerts R de Girolamo G Gureje O Huang Y Karam A Kostyuchenko S Lepine J Mora M Neumark Y Ormel JH Pinto-Meza A Posada-Villa J Stein D Takeshima T Wells JE Toward a global view of alcohol, tobacco, cannabis, and cocaine use: Findings from the WHO World Mental Health Surveys PLoS Med 2008 5 7 1053 1065 10.1371/journal.pmed.0050141 PMC2443200 18597549

[B32] Heo M Leon AC Sample size requirements to detect an intervention by time interaction in longitudinal cluster randomized clinical trials Stat Med 2009 28 1017 1027 19153969 10.1002/sim.3527 PMC2758777

[B33] Teesson M Newton NC Barrett E Australian school-based prevention programs for alcohol and other drugs: A systematic review Drug Alcohol Rev 2012 31 6 731 736 22340636 10.1111/j.1465-3362.2012.00420.x

[B34] Nherera L Jacklin P A model to assess the cost-effectiveness of alcohol education developed for NICE public health guidance on personal, social, health and economic (PSHE) education. In 2009 London: National Collaborating Centre for Women's and Children's Health

[B35] Hansen WB School-based substance abuse prevention: A review of the state of the art in curriculum, 1980–1990 Health Educ Res 1992 7 3 403 430 10171672 10.1093/her/7.3.403

[B36] Tobler NS Stratton HH Effectiveness of School-based drug prevention programs: A meta-analysis of the research J Prim Prev 1997 18 1 71 128

[B37] Tobler NS Roona MR Ochshorn P Marshall DG Streke AV Stackpole KM School-based adolescent drug prevention programs: 1998 meta-analysis J Prim Prev 2000 20 4 275 336

[B38] Botvin G Griffin K Sloboda Z, Bukoski WJ Drug abuse prevention curricula in schools Handbook of drug abuse prevention: Theory, science and practice. edn 2003 New York: Kluwer Academic/Plenum Publishers

[B39] Williams C Perry C Lessons from Project Northland: Preventing alcohol problems during adolescence Alcohol Health Res World 1998 22 2 107 116 15706784 PMC6761807

[B40] McBride N Farringdon F Midford R Meuleners L Phillips M Hill L Hamilton G Roche A Anderson P Harm minimisation in school drug education: Final results of the School Health and Alcohol Harm Reduction Project (SHAHRP). Commentary Addiction 2004 99 3 278 298 14982537 10.1111/j.1360-0443.2003.00620.x

[B41] Newman I Anderson C Farrell K Role rehearsal and efficacy: Two 15 month evaluations of a ninth grade alcohol education programme J Drug Educ 1992 22 55 67 1593388 10.2190/QQWE-PFJ5-PVKY-FQQK

[B42] McBride N A systematic review of school drug education Health Educ Res 2003 18 6 729 742 14654505 10.1093/her/cyf050

[B43] Tobler NS Meta-analysis of 143 adolescent drug prevention programs: Quantitative outcome results of program participants compared to a control or comparison group Journal of Drug Issues 1986 16 4 537 567

[B44] Hansen W Program evaluation strategies for substance abuse prevention J Prim Prev 2002 22 4 409 436

[B45] Hingson RW Heeren T Winter MR Age at drinking onset and alcohol dependence: Age at onset, duration and severity Arch Pediatr Adolesc Med 2006 160 739 746 16818840 10.1001/archpedi.160.7.739

[B46] Behrendt S Wittchen H Hofler M Lieb R Beesdo K Transitions from first substance use to substance use disorders in adolescence: Is early onset associated with a rapid escalation? Drug Alcohol Depend 2009 99 68 78 18768267 10.1016/j.drugalcdep.2008.06.014

[B47] Swift W Coffey C Carlin JB Degenhardt L Calabria B Patton G Are adolescents who moderate their cannabis use at lower risk of later regular and dependent cannabis use? Addiction 2009 104 806 814 19344439 10.1111/j.1360-0443.2009.02534.x

[B48] Rohrbach L Milam J Sloboda Z, Bukoski WJ Gender Issues in substance abuse prevention Handbook of Drug Abuse Prevention: Theory, Science and Practice. edn 2003 New York: Kluwer academic/Plenum Publishers

[B49] von Sydow K Lieb R Pfister H Hofler M Sonntag H Wittchen H The natural course of cannabis use, abuse and dependence over four years: a longitudinal community study of adolescents and young adults Drug Alcohol Depend 2001 64 347 361 11672949 10.1016/s0376-8716(01)00137-5

[B50] Swift W Coffey C Carlin JB Degenhardt L Patton G Adolescent cannabis users at 24 years: trajectories to regular weekly use and dependence in young adulthood Addiction 2008 103 1361 1370 18855826 10.1111/j.1360-0443.2008.02246.x

[B51] Nolen-Hoeksema S Hilt L Possible contributors to the gender differences in alcohol use problems J Gen Psychol 2006 133 4 357 374 17128956 10.3200/GENP.133.4.357-374

[B52] Swift W Copeland J Treatment needs and experiences of Australian women with alcohol and other drug problems Drug Alcohol Depend 1996 40 211 219 8861399 10.1016/0376-8716(95)01209-5

[B53] Stoltenberg SC Batien BD Birgenheir DG Does gender moderate associations among impulsivity and health-risk behaviours? Addict Behav 2008 33 252 265 17913380 10.1016/j.addbeh.2007.09.004 PMC2225595

[B54] K-Y LIANG ZEGER SL Longitudinal data analysis using generalized linear models Biometrika 1986 73 1 13 22

[B55] Twisk JW Applied longitudinal data analysis for epidemiology: a practical guide 2013 Cambridge: Cambridge University Press

[B56] Botvin G Griffin K Diaz T Ifill-Williams M Preventing binge drinking during early adolescence: One- and two- year follow-up of a school-based preventive intervention Behaviors Psychol Addict Behav 2001 15 4 360 365 10.1037//0893-164x.15.4.360 11767269

[B57] McBride N Farringdon F Midford R Meuleners L Phillips M Harm minimization in school drug education: Final results of the School Health and Alcohol Harm Reduction Project (SHAHRP) Addiction 2004 99 3 278 291 14982537 10.1111/j.1360-0443.2003.00620.x

[B58] Biglan A Steverson H Ary D Faller C Gallison C Thompson R Glasgow R Lichtenstein E Do smoking prevention programs really work? Attrition and the internal and external validity of an evaluation of refusal skills training program J Behav Med 1987 10 2 159 171 3612776 10.1007/BF00846424

[B59] Ellickson PL McCaffrey DF Ghosh-Dastidar B Longshore DL New inroads in preventing adolescent drug use: Results from a large-scale trial of Project ALERT in middle schools Am J Public Health 2003 93 11 1830 1836 14600049 10.2105/ajph.93.11.1830 PMC1448059

[B60] Winters KC Monti PM, Colby SM, O'Leary TA Assessing adolescent substance use problems and other areas of functioning Adolescents, Alcohol and Substance Abuse: Reaching Teens through Brief Interventions. edn 2001 New York: The Guilford Press

[B61] Winters KC Stinchfield RD Henly GA Schwartz R Validity of adolescent self-report of alcohol and other drug involvement Int J Addict 1990–1991 25 11A 1379 1395 2132719 10.3109/10826089009068469

[B62] Donaldson SI Thomas CW Graham JW Au JG Hansen WB Verifying drug abuse prevention program effects using reciprocal best friend reports J Behav Med 2000 23 6 585 601 11199089 10.1023/a:1005559620739

[B63] Winchester L Dobbinson S Rissel C Bauman A Anonymous record linkage using respondent-generated identification codes - a tool for health promotion research Health Promot J Austr 1996 6 2 52 54

[B64] Champion KE Newton NC Barrett EL Teesson M A systematic review of school-based alcohol and other drug prevention programs facilitated by computers or the Internet Drug Alcohol Rev 2013 32 2 115 123 23039085 10.1111/j.1465-3362.2012.00517.x

[B65] Conrod PJ Castellanos N Mackie C Personality-targeted interventions delay the growth of adolescent drinking and binge drinking J Child Psychol Psychiatry 2008 49 2 181 190 18211277 10.1111/j.1469-7610.2007.01826.x

[B66] Conrod PJ Castellanos-Ryan N Strang J Brief, personality-targeted coping skills interventions and survival as a non-drug user over a 2-year period during adolescence Arch Gen Psychiatry 2010 67 1 85 93 20048226 10.1001/archgenpsychiatry.2009.173

